# Attitudes toward Nutrition Care among General Practitioners in Croatia

**DOI:** 10.3390/jcm7040060

**Published:** 2018-03-21

**Authors:** Albina Dumic, Ivan Miskulin, Nika Pavlovic, Daniela Cacic Kenjeric, Zelimir Orkic, Maja Miskulin

**Affiliations:** 1 Faculty of Medicine, Josip Juraj Strossmayer University of Osijek, 31 000 Osijek, Croatia; albina.dumic@gmail.com (A.D.); ivan.miskulin@mefos.hr (I.M.); nika.pavlovic@mefos.hr (N.P.); zelimir.orkic@gmail.com (Z.O.); 2 Faculty of Food Technology, Josip Juraj Strossmayer University of Osijek, 31 000 Osijek, Croatia; daniela.kenjeric@ptfos.hr

**Keywords:** nutrition, attitudes, general practitioners, primary health care, Croatia

## Abstract

Nutrition care should be an integral part of general practitioners’ (GPs’) daily work with patients. The aim of this study was to assess the attitudes of Croatian GPs toward nutrition and nutrition care, and to evaluate the interconnection between their attitudes and implementation of nutrition care in GPs offices. A cross-sectional study was conducted among 17.0% of randomly selected GPs, from May to July of 2013, via a specially designed anonymous questionnaire. The study showed that 36.0% of the Croatian GPs had satisfactory number of positive attitudes (5 or more) toward nutrition and nutrition care. There was statistically significant difference in the median number of positive attitudes based on the additional education of GPs in nutrition and their ailment from chronic diseases (*p <* 0.001 and *p* = 0.022, respectively). The Spearman rank correlation between GPs’ attitudes toward nutrition and nutrition care and their practice, i.e., the implementation of nutrition care in GPs’ everyday work with patients was *r*_s_ = −0.235 (*p <* 0.001). In order to provide nutrition care in GPs’ offices in Croatia, strategies for changing GPs’ attitudes toward nutrition and nutrition care are needed.

## 1. Introduction

Poor nutrition is one of the most dominant lifestyle-related risk factors for chronic diseases [1,2] and has also been shown to have adverse effects on acute illnesses [3]. Epidemiological data show that seven out of the ten leading causes of death are chronic diseases that possess a critical nutrition component, such as heart disease, cancer and diabetes [4,5,6].

The World Health Organization has previously recommended that medical professionals be supported in taking an active role in promoting healthy nutritional behaviors and providing nutrition care [7]. General practitioners (GPs) provide coordinated holistic health care to individuals and families in their communities [8]. GPs are therefore positioned in contact with people, and patients expect their physicians to provide them with health information [9]. They are a trusted source of advice on a range of issues, including nutrition [1,10,11]. Furthermore, it has been shown that 70% of the general public visit their GP at least one time each year [10]. Health workers in primary health care settings are particularly important providers of nutrition care, because they can motivate even healthy individuals to adopt healthier lifestyles [5]. Furthermore, the primary health care setting has been identified as an ideal setting for implementing chronic disease management programs, including the provision of nutrition care [1,12,13].

Nutrition care refers to any practice undertaken by a health professional to improve an individual’s food-related behavior and subsequent health outcomes [1,2,6,14,15,16]. Studies have shown that GPs can be very effective in enhancing patients’ nutrition behavior through the implementation of nutrition care [17,18]. However, the delivery of nutrition care by doctors has been reported to be quite rare [13,18]. Studies have shown that physicians attitudes towards nutrition and nutrition care are significant predictors of physicians’ daily nutrition practice [19,20]. Thus, the aim of this study was to explore the attitudes of Croatian GPs toward nutrition and nutrition care, and to evaluate the interconnection between their attitudes and implementation of nutrition care in GPs’ offices.

## 2. Materials and Methods

### 2.1. Study Population and Survey

From among the doctors who work in primary health care offices in Croatia and are registered in the register of GPs managed by the Croatian National Institute of Public Health, 800 potential participants (30.6%) were chosen by random selection and were sent an anonymous questionnaire via post. Along with the questionnaire, each GP, a potential participant, received an explanation of the study, an informed consent form and two envelopes containing the address of the lead author. They were asked to sign the consent form before answering the questionnaire and to put the signed informed consent form in one envelope, and the answered questionnaire in the other envelope and then send them to the lead author. Thereby, the anonymity of the study was ensured and by no means could the personal data, specifically the name and surname, of the participants be connected to the answers provided in the questionnaire. The answered questionnaires that arrived via post were assigned codes, and the data provided within the questionnaires were later analyzed using that code. The response rate was 55.5% (444/800). The study included 17.0% (444/2.612) of doctors who work in primary health care offices in Croatia, and the study participant sample was representative for this population of Croatian doctors. The described cross-sectional study was conducted from 1 May to 31 July 2013. The study was approved by the Ethics Committee of the Faculty of Medicine Osijek, Croatia (Ethical Approval Code: 2158-61-07-12-35).

### 2.2. Questionnaire

This study was conducted through an anonymous self-administered questionnaire composed of 30 questions divided into four sections. The first section of the questionnaire (six questions) referred to the sociodemographic characteristics of the study participants such as age, gender, education, length of service, additional education in the field of nutritional science, and ailments from chronic diseases where improper nutrition poses as a risk factor. The second section of the questionnaire (ten questions) referred to the GP’s knowledge of the field of nutritional science. The third section of the questionnaire (ten questions) involved the attitudes the GPs had toward the significance of the nutrition in the treatment and prevention of chronic diseases and toward the nutrition care while the fourth section of the questionnaire (four questions) referred to the nutrition counselling practice of GPs in their daily work with patients. The questionnaire used in this study was previously validated with a smaller group of participants in 2012. Within this paper, questions about the sociodemographic characteristics of the study participants, questions about the GPs’ attitudes toward the significance of the nutrition in the treatment and prevention of chronic diseases and their attitudes toward the nutrition care as well as question regarding the implementation of nutrition care in the GPs’ everyday work with patients were analyzed. In the analysis of a satisfactory number of positive attitudes, a cut-off value of 5 positive attitudes was applied, because it has been shown that positive attitudes of 50% or more are sufficient to ensure acceptable nutrition care in GPs offices.

### 2.3. Statistical Analysis

Upon confirming the normality of the data distribution by the Kolmogorov-Smirnov test, all data were processed by the methods of descriptive statistics. The numerical variables were described as median and interquartile range. The Mann-Whitney U test was used for the comparison of numerical variables among the groups. The categorical variables were described in absolute and relative frequencies. The χ^2^-test was used for the comparison of categorical variables between the groups. The Spearman’s correlation coefficient (*r*_s_) was calculated to test the correlation between the implementation of nutrition care in GPs everyday work with patients and their attitudes toward nutrition and nutrition care. The level of statistical significance was set at *p <* 0.05. Statistical analysis was done using the statistical package Statistica for Windows 2010 (version 10.0, StatSoft Inc., Tulsa, OK, USA).

## 3. Results

A total of 444 participants (81.3% of females and 18.7% of males) were included in the study. The mean age of all the participants was 50 years old, ranging from 25 to 67 years. Among all the participants, 67.3% were specialists in family medicine and 32.7% were doctors who had licenses for an independent practice, but without a finished specialization in the field of family medicine. According to the length of service, 65.1% of the participants had 15 or more and 34.9% had 0–14 years of service. Among the participants, 9.5% had completed additional educational programs in the field of nutritional science and 30.6% had suffered from some chronic disease with unbalanced nutrition posing as a risk factor. Among all participants, there were 36.0% with a satisfactory number (5 or more) of positive attitudes toward the significance of nutrition in the treatment and prevention of chronic diseases and toward nutrition care, and 64.0% with an unsatisfactory number (4 or less) of positive attitudes.

Table 1 shows the Croatian GPs with and without a satisfactory number of positive attitudes toward the significance of the nutrition in the treatment and prevention of chronic diseases and toward nutrition care according to their sociodemographic characteristics.

The median number of positive attitudes held by Croatian GPs toward the significance of nutrition in the treatment and prevention of chronic diseases and toward nutrition care was 3.00 (interquartile range 3.00–9.00). There was no statistically significant difference in the median number of positive attitudes between males and females (*p* = 0.337), between GPs with 14 or less and GPs with 15 or more years of experience (*p* = 0.381), between younger GPs (45 years old or less) and older GPs (46 years old or more) (*p* = 0.163), or between specialists of family medicine and GPs without specialization (*p* = 0.578). The study revealed that the GPs with additional education in nutrition and the GPs who had not been suffering from a chronic disease had more positive attitudes toward nutrition and nutrition care (*p <* 0.001 and *p* = 0.022, respectively).

Table 2 shows attitudes of the Croatian GPs toward the significance of nutrition in the treatment and prevention of chronic diseases according to the sociodemographic characteristics of study participants.

Table 3 shows attitudes of Croatian GPs toward nutrition care according to the sociodemographic characteristics of study participants.

The study showed that during their everyday work with patients, 95.7% of Croatian GPs provide nutrition care, while 4.3% of them do not provide such care at all. Among those who provide nutrition care, there were 19.5% who provide nutrition care for all patients regardless of their individual health risks and 80.5% who provide such care only for patients considered at risk with regard to their bad nutritional habits and/or elevated body mass index.

The Spearman rank correlation between GPs attitudes toward nutrition and nutrition care and their practice, i.e., the implementation of nutrition care in GPs everyday work with patients, was *r*_s_ = −0.235 (*p <* 0.001).

## 4. Discussion

In this paper, we assessed attitudes of Croatian GPs toward the significance of nutrition in the treatment and prevention of chronic diseases and toward nutrition care and the implementation of nutrition care in their everyday work with patients. The study showed that only 36.0% of Croatian GPs had a satisfactory number of positive attitudes (5 or more) toward investigated variables, pointing to the rather unsatisfactory situation regarding the researched issues within the study population. The determined proportion of Croatian GPs with a satisfactory number of positive attitudes was much lower than the proportions of GPs determined in similar studies conducted in New Zealand, Taiwan, the USA, and the UK, where the percentage of positive attitudes toward nutritional statements were above 90%, above 95%, 82%, and 99%, respectively [2,9,21,22].

The present study showed that there was no statistically significant difference in the proportion of Croatian GPs with and without a satisfactory number of positive attitudes toward the significance of the nutrition in the treatment and prevention of chronic diseases and toward the nutrition care between males and females, between GPs with 14 or less and GPs with 15 or more years of experience, between younger GPs (45 years old or less) and older GPs (46 years old or more), or between specialists of family medicine and GPs without specialization. Some of these results are different when compared to the results of similar studies conducted elsewhere. For example, studies conducted in Taiwan, USA and Canada found that the investigated attitudes varied with age, with younger GPs tending to have more positive attitudes [9,21,23]. However, some of the results are similar to the results of other studies. As in this study, a study conducted in New Zealand did not find a difference in the proportion of positive attitudes between doctors with a specialization in family medicine and doctors without the specialization [2].

The present study revealed that additional education in nutrition and personal ailment from chronic diseases significantly influences the GPs’ attitudes toward the significance of nutrition in the treatment and prevention of chronic diseases and toward nutrition care. The connection between additional education in nutrition and physicians’ attitudes was also detected in study conducted among GPs in the USA [21]. The latter connection has been further confirmed in recent studies that proved that additional training in nutrition can facilitate positive changes in physicians’ attitudes toward nutrition care [24,25,26]. Regarding the personal ailment from chronic diseases and attitudes toward the significance of nutrition in the treatment and prevention of chronic diseases and toward nutrition care, it seems that Croatian GPs who have already suffered from chronic diseases with improper nutrition posing as a risk factor, despite their illness, still do not recognize the significance of nutritional factors in their etiology. The latter is quite disturbing since GPs having less favorable personal attitudes toward nutrition is bad model for their patients, who perceive GPs as a trusted source of advice on a range of issues, including nutrition [10]. It is well known that those physicians who modified their own nutrition were more likely to apply nutrition care in their practice. This fact can probably explain why those Croatian GPs who had not suffered from chronic diseases with improper nutrition posing as a risk factor had more positive attitudes toward the significance of nutrition in the treatment and prevention of chronic diseases and toward nutrition care [21].

The study showed that during their daily everyday work with patients, 95.7% of Croatian GPs provided nutrition care, but they predominantly practice a therapeutic approach, since of these, 80.5% provided such care only for patients considered at risk with regard to their bad nutritional habits and/or elevated body mass index, and only 19.5% provided nutrition care for all patients regardless of their individual health risks. This situation is similar to the situation discovered in the study conducted among GPs in Saudi Arabia, where physicians also seemed to identify nutritional problems as part of chronic disease care more often than for screening purposes, which suggests a therapeutic rather than preventive approach toward such problems [27].

Considering the interconnection between Croatian GPs’ attitudes toward nutrition and nutrition care and the implementation of nutrition care in their offices, this study revealed that despite the very modest percentage of GPs with positive attitudes toward nutrition and nutrition care of only 36%, over 95% of Croatian GPs provide nutrition care to their patients in everyday practice. Studies conducted in Australia, Saudi Arabia, and the USA have also pointed to the noticeable gap that exists between physicians’ attitudes and their current practice with regard to nutrition and nutrition care, but in these studies, patients did not receive nutrition care despite the largely positive attitudes of their GPs [8,27,28]. Considering that, the situation in Croatia can be viewed as not so critical, because the most important thing is that patients eventually get nutrition care, although room for improvement certainly exists, especially in the reorientation of such care toward primary prevention, as already mentioned [13]. Contrary to the aforementioned studies, studies in Taiwan and Canada have shown that physicians attitudes were strongly associated with providing nutrition care [9,23].

The findings of this study significantly increase our understanding regarding the Croatian GPs’ attitudes toward nutrition and nutrition care. Our evaluation is based on the nationally representative sample of Croatian GPs, with a high representation of different age cohorts of GPs. As previously emphasized, 17.0% of doctors who work in primary health care offices in Croatia had been questioned for this study, so their answers can be considered representative.

However, our study is not without limitations. Its cross-sectional design makes it difficult to establish causality. Nonetheless, it gives a clear snapshot of the attitudes and practice of Croatian GPs with regard to nutrition and nutrition care. As with all self-administered studies, the present study was subject to volunteer bias, although the effects of this are minimized by the response rate of nearly 60%. Furthermore, self-reported data do not always reflect daily clinical practice, and attitudes are often influenced by the daily workload in practice and physicians’ actual mood. Results might also have been subject to a social desirability response bias, whereby respondents give socially desirable and acceptable responses rather than reporting their actual attitudes or practice. Despite latter observation, it is widely accepted that self-report measures of nutrition-related competences could be used as a proxy for actual measures of competence. In addition, it was established that findings of self-reports are usually as valid as more elaborate and expensive tests [11].

Future studies should explore factors contributing to the current status of GPs’ attitudes toward nutrition care in Croatia, especially those connected with the general situation within the Croatian health system. Also, future studies should consider the attitudes of future doctors and its interconnection with the place that nutrition, nutrition care and public health in general have within the curricula of medical faculties in Croatia.

In conclusion, one can say that there is a rather modest percentage of Croatian GPs with positive attitudes toward nutrition and nutrition care and no interconnection between GPs’ attitudes and implementation of nutrition care in their offices. In order to increase the proportion of Croatian GPs with positive attitudes, and also to empower the Croatian GPs in their nutrition care practices, it is necessary to design and implement health policy measures that are predominantly oriented toward prevention.

## Figures and Tables

**Table 1:** The Croatian GPs with and without a satisfactory number of positive attitudes toward the significance of nutrition in the treatment and prevention of chronic diseases and toward nutrition care according to their sociodemographic characteristics.

Participant Characteristics	Number of Participants (%)			*p* *
	With Satisfactory Number of Positive Attitudes (5 or More)	Without Satisfactory Number of Positive Attitudes (4 or Less)	Overall	
**Gender**				
Male	22 (13.7)	61 (21.5)	83 (18.7)	0.057
Female	138 (86.3)	223 (78.5)	361 (81.3)	
**Length of service (years)**				
0–14	50 (31.2)	105 (37.0)	155 (34.9)	0.254
15 or more	110 (68.8)	179 (63.0)	289 (65.1)	
**Age group (years)**				
Younger (45 or less)	52 (32.5)	114 (40.1)	166 (37.4)	0.126
Older (46 or more)	108 (67.5)	170 (59.9)	278 (62.6)	
**Education**				
Doctors with a license for independent practice without a finished specialization	58 (36.3)	87 (30.6)	145 (32.7)	0.247
Specialist in family medicine	102 (63.7)	197 (69.4)	299 (67.3)	
**Additional education about nutrition**				
Yes	35 (21.9)	7 (2.5)	42 (9.5)	<0.001
No	125 (78.1)	277 (97.5)	402 (90.5)	
**Suffering from chronic diseases**				
Yes	35 (21.9)	101 (35.6)	136 (30.6)	0.003
No	125 (78.1)	183 (64.4)	308 (69.4)	
**Overall**	160 (36.0)	284 (64.0)	444 (100.0)	

* χ^2^-test.

**Table 2:** Attitudes of the Croatian GPs toward the significance of nutrition in the treatment and prevention of chronic diseases according to the sociodemographic characteristics of study participants.

Participant Characteristics	Number of Positive Attitudes		*p* *
	Median (25–75%)	Min—Max	
**Gender**			
Male	3.00 (3.00–4.00)	2.00–5.00	0.075
Female	3.00 (3.00–4.00)	2.00–5.00	
**Length of service (years)**			
0–14	3.00 (3.00–5.00)	1.00–5.00	0.486
15 or more	3.00 (3.00–5.00)	1.00–5.00	
**Age group (years)**			
Younger (45 or less)	3.00 (3.00–5.00)	1.00–5.00	0.223
Older (46 or more)	3.00 (3.00–5.00)	1.00–5.00	
**Education**			
Doctors with a license for independent practice without a finished specialization	3.00 (3.00–5.00)	2.00–5.00	0.300
Specialist in family medicine	3.00 (3.00–5.00)	1.00–5.00	
**Additional education about nutrition**			
Yes	5.00 (4.00–5.00)	2.00–5.00	<0.001
No	3.00 (3.00–5.00)	1.00–5.00	
**Suffering from chronic diseases**			
Yes	3.00 (3.00–3.75)	2.00–5.00	0.009
No	3.00 (3.00–5.00)	1.00–5.00	
**Overall**	3.00 (3.00–5.00)	1.00–5.00	

* Mann-Whitney U test.

**Table 3:** Attitudes of Croatian GPs toward the nutrition care according to the sociodemographic characteristics of study participants.

Participant Characteristics	Number of Positive Attitudes		*p* *
	Median (25–75%)	Min–Max	
**Gender**			
Male	0.00 (0.00–4.00)	0.00–5.00	0.323
Female	0.00 (0.00–4.00)	0.00–5.00	
**Length of service (years)**			
0–14	0.00 (0.00–4.00)	0.00–5.00	0.145
15 or more	0.00 (0.00–4.00)	0.00–5.00	
**Age group (years)**			
Younger (45 or less)	0.00 (0.00–4.00)	0.00–5.00	0.416
Older (46 or more)	1.00 (0.00–4.00)	0.00–5.00	
**Education**			
Doctors with a license for independent practice without a finished specialization	0.00 (0.00–4.00)	0.00–5.00	0.610
Specialist in family medicine	0.00 (0.00–4.00)	0.00–5.00	
**Additional education about nutrition**			
Yes	4.00 (3.00–5.00)	0.00–5.00	<0.001
No	0.00 (0.00–4.00)	0.00–5.00	
**Suffering from chronic diseases**			
Yes	0.00 (0.00–2.75)	0.00–5.00	0.024
No	1.00 (0.00–4.00)	0.00–5.00	
**Overall**	0.00 (0.00–4.00)	0.00–5.00	

* Mann-Whitney U test.
